# “Rogue” [DEspR+CD11b+] neutrophil subset correlates with severity in spontaneous intracerebral hemorrhage

**DOI:** 10.3389/fneur.2022.935579

**Published:** 2022-07-25

**Authors:** Victoria L. M. Herrera, Courtney E. Takahashi, Mai Q. Nguyen, Julie Z. Mosaddeghi, Ridiane Denis, David M. Greer, Nelson Ruiz-Opazo

**Affiliations:** ^1^Whitaker Cardiovascular Institute and Department of Medicine, Boston University School of Medicine, Boston, MA, United States; ^2^Department of Neurology, Boston Medical Center and Boston University School of Medicine, Boston, MA, United States; ^3^General Clinical Research Unit, Boston University School of Medicine, Boston, MA, United States

**Keywords:** spontaneous intracerebral hemorrhage, neutrophil subsets/heterogeneity, secondary brain injury, DEspR+CD11b+ neutrophils, NETs (neutrophil extracellular traps)

## Abstract

**Objective:**

Cumulative clinical, cellular, and molecular evidence reinforces the role of neutrophils in secondary brain injury in spontaneous intracerebral hemorrhage (sICH). However, since generalized neutrophil inhibition is detrimental, identification of targetable “rogue” neutrophil subsets associated with sICH severity is key.

**Methods:**

In a pilot prospective observational study of consented patients with sICH, we immunotyped whole blood to assess circulating neutrophil markers (~day 3 after ICH symptoms onset): (a) DEspR±CD11b± neutrophils by flow cytometry, (b) DEspR±CD11b± neutrophil extracellular trap (NET)-forming neutrophils by immunofluorescence cytology, and (c) neutrophil-lymphocyte ratio (NLR). Using Spearman rank correlation (*r*) with Bonferroni correction, we assessed the association of neutrophil markers with same-day clinical and neuroimaging parameters of sICH severity, index ICH score, 90-day modified Rankin Scale (mRS) score, and potential interrelationships. As comparators, we assessed same-day plasma biomarkers elevated in sICH: interleukin-6/IL-6, myeloperoxidase/MPO, soluble-terminal complement complex/sC5b-9, endothelin-1/ET-1, and mitochondrial/nuclear DNA ratio (mt/nDNA ratio).

**Results:**

We detected strong correlations [*r*(n = 13) > 0.71, power > 0.8, Bonferroni corrected *p*^*B*^ < 0.05] for all three neutrophil markers with 90-day mRS score, differentially for DEspR+CD11b+ neutrophil counts, and NLR with perihematomal edema (PHE) volume and for DEspR+CD11b+ NET-forming neutrophil counts with intraparenchymal hemorrhage (IPH)-volume. Only DEspR+CD11b+ neutrophil counts show a strong correlation with index ICH score, same-day Glasgow Coma Scale (GCS) score, and NLR and NET-forming neutrophil counts. The sum of the ICH score and three neutrophil markers exhibited the highest correlation: [*r*(n = 13) 0.94, *p*^*B*^ = 10^−5^]. In contrast, plasma biomarkers tested were elevated except for MPO but exhibited no correlations in this pilot study.

**Conclusion:**

Strong correlation with multiple sICH severity measures, NET formation, and NLR identifies DEspR+CD11b+ neutrophils as a putative “rogue” neutrophil subset in sICH. The even stronger correlation of the sum of three neutrophil markers and the index ICH score with 90-day mRS outcome reinforces early neutrophil-mediated secondary brain injury as a key determinant of outcome in patients with sICH. Altogether, data provide a basis for the formal study of the DEspR+CD11b+ neutrophil subset as a potential actionable biomarker for neutrophil-driven secondary brain injury in sICH. Data also show *ex vivo* analysis of patients with sICH neutrophils as a translational milestone to refine hypotheses between preclinical and clinical studies.

## Introduction

Spontaneous intracerebral hemorrhage has the highest mortality among all stroke subtypes and remains without FDA-approved pharmacotherapy to reduce morbidity and mortality (1). The high mortality is due, at least in part, to neuroinflammation-mediated secondary brain injury manifesting as perihematomal edema (2). As the reduction of the primary injury by hematoma evacuation has not improved mortality in sICH, albeit safe (3), reduction of feed-forward secondary brain injury becomes key to improving outcomes in sICH (1, 2).

Although neuroinflammation is complex and multifactorial, cumulative data support the key roles of neutrophils in secondary brain injury in sICH (4) and systemic inflammation associated with poor outcomes in sICH (5). First, recent histopathology staining of postmortem sICH brain sections has confirmed neutrophil presence in the perihematomal region starting day1 post-ICH (6) and neutrophil extracellular trap (NET)-forming neutrophils at least by day 3 (7). Second, recent transcription profile analyses report that circulating neutrophils in patients with sICH differ in gene expression compared with age-matched non-ICH controls (8). Third, neutrophil-specific gene changes in the sICH brain and peripheral neutrophils are similar, indicating the pathogenic relevance of studying peripheral neutrophils (8). Fourth, a meta-analysis of multiple clinical studies confirmed the association of high NLR with worse outcomes in sICH (9), further supported by a recent study showing the association of elevated NLR with poor outcomes and perihematomal edema (10). Altogether, these data reiterate the central role of neutrophils in sICH secondary brain injury and along with emerging evidence of neutrophil heterogeneity (11), delineate the importance of determining neutrophil subset-specific roles in secondary brain injury in sICH.

We recently identified the DEspR+CD11b+ “rogue” neutrophil subset associated with mortality and worse sequential organ failure assessment (SOFA) scores in patients with acute respiratory distress syndrome (ARDS), the prime example of feed-forward neutrophil-mediated tissue injury (12). “Rogue” DEspR+CD11b+ neutrophils exhibit delayed apoptosis, which is inhibited by a humanized, anti-DEspR antibody, hu6g8, in *ex vivo* studies (12). Concordantly, in a hypertensive, spontaneous ICH (hsICH) rat model, we detected DEspR+ neutrophils in perihematomal brain regions with evidence of blood-brain barrier (BBB) disruption at acute sICH (13). Most importantly, both murine anti-rat DEspR and humanized anti-rat/human DEspR antibody treatments at acute sICH improved median survival of hsICH-rats, with an empirical resolution of presenting neurological symptoms and without an increased infection risk (14).

Altogether, these observations delineate the hypothesis that an increased DEspR+CD11b+ neutrophil subset contributes to the progression of neutrophil-mediated secondary brain injury and is thus associated with early mortality and worse outcomes in patients with sICH. Herein, we tested this hypothesis in a prospective pilot observational study *via ex vivo* analysis of patients with sICH peripheral whole blood by flow cytometry, immunofluorescence-cytology, and plasma biomarker characterization of the intravascular proinflammatory milieu. We aimed to (1) demonstrate the clinical feasibility of immunotyping for the DEspR+CD11b+ neutrophil subset in patients with sICH and (2) determine the association of the DEspR+CD11b+ neutrophil subset with clinical and radiological measures of sICH severity compared with emerging biomarkers associated with sICH severity, to gain insight into their potential as therapeutic targets whose inhibition could attenuate feed-forward secondary brain injury in sICH.

## Methods

### Blinded observational study design

No patient samples with sICH obtained from consented subjects were excluded. The clinical and basic science researchers were blinded to the data of the other. Whole blood samples were collected and analyzed, and results were recorded in data sheets blinded to sICH severity measures. Likewise, sICH clinical and radiological severity measures were obtained blinded to data from flow cytometry, immunofluorescence staining, and ELISA measurements. More specifically, the different tasks in this interdisciplinary pilot observational study were compartmentalized to attain blinding of researchers during task performance. The following tasks were compartmentalized: (a) patient screening, (b) consenting and blood sampling, (c) processing of blood for flow cytometry and FlowJo analysis, (d) clinical data collection, (e) laboratory testing – ELISAs, (f) preparation of blood smears from whole blood, (g) immune-fluorescence staining, (h) confocal microscopy imaging and semiquantitative measures, and (i) analysis of collated laboratory and clinical data.

### Study subjects

All subjects were identified in the ICU following a study protocol approved by the Institutional Review Board (IRB) of Boston University and Boston Medical Center (IRB H-38089). Each subject's legally authorized representative (LAR) gave written informed consent for study participation in compliance with the Declaration of Helsinki. We prospectively enrolled 13 patients with sICH.

Inclusion criteria: Adult patients (>18 to < 90 years) with sICH admitted to the ICU with ICH diagnosis by noncontrast head computed tomography (CT)-scans.

Exclusion criteria: ICH secondary to trauma, aneurysm, vascular malformation, or neoplasm; patients who are prisoners, pregnant women due to immunological changes during pregnancy, mRS > 1 prior to enrollment, patients on chemotherapy or immunotherapy, or with infections requiring BSL-3 containment were also excluded.

Clinical information obtained included age, gender, race, medical history of hypertension or hyperlipidemia, complete blood count (CBC) with differential, ICH scores (calculated with age, admission GCS, hemorrhage location, intraventricular extension, and hematoma volume > 30 cc), Glasgow Coma Scale (GCS), modified Rankin Scale (mRS) score at the end of ICU stay, at discharge from hospital, and at 90-day post-ICH. Radiological parameters, including hematoma volumes and perihematomal edema volumes, modified Graeb scores (maximum score 32), measured based on noncontrast head CT, were collected and entered into a HIPAA-compliant data sheet for further analysis.

### Quantification of radiological parameters

Intraparenchymal hemorrhage, perihematomal edema (PHE) volumes, and modified Graeb scores were calculated from CT scan on the day of flow cytometry ± 1 day by two board-certified neurointensivists (CT and DG).

#### Hematoma volume quantification

Hematoma volumes (IPH) were calculated using the ABC/2 method using axial, non-contrast head CT with 0.5 cm slices. In this methodology, the rater visually inspects each slice of head CT and selects the 0.5 cm slice with the largest length and width orthogonal to the other. The length (A) and width (B) are measured in cm using the embedded ruler tool in the Picture Archiving and Communications Systems (PACS) Radiology software (www.vitalimages.com). Slices with < 25% hemorrhage are not counted. Calculated volume (cc) is equal to [length (A) × width (B) × the number of slices (C)] divided by 2 to compensate for the ellipsoidal hematomal volume.

#### Perihematomal edema volume quantification

The same ABC/2 methodology described above was used to estimate perihematomal edema volumes. Instead of measuring the length and width with hemorrhage borders, the edema, which is hypodense when compared with the rest of the parenchyma, was used. To calculate the edema volume, the hematoma volumes were subtracted.

#### Computer volume quantification

A semiautomated IPH and PHE volume estimates were generated using Python (Python Software Foundation. Python Language Reference, version 3.8. Available at http://www.python.org). The program utilizes the principles of dual-clustering segmentation for soft tissue identification—white matter, gray matter, hemorrhage, and edema—which interrogates each voxel to determine whether it belongs in a user predefined Hounsfield unit subvolume and whether its surrounding pixels were similarly radio dense. Segmentations were improved through the assessment of pixel cluster internal connectivity and pixel cluster intensity.

Image processing consists of two steps: automated segmentation and volume calculation. After loading the CT dataset, the total brain tissue is extracted from the skull *via* dual-clustering and connected component segmentation. The ICH is similarly segmented from the brain tissue based on elevated radio density. The perihematomal edema pixels were identified by successive three-dimensional dilation of the hemorrhage in binary format to a user-defined limit. The hemorrhage core is then subtracted from the IPH-PHE volume, and the PHE-only pixels are refined through a third dual-clustering segmentation step utilizing edema-specific Hounsfield units. The *in toto* brain, IPH, and PHE volumes are calculated by multiplying the total number of pixels in each segment by the known voxel size.

All ICH and PHE values were recorded into a database.

### Blood collection

Whole blood (6 ml) was collected *via* preexisting indwelling peripheral vascular lines into K2-EDTA vacutainer tubes (FisherScientific, MA, USA). Blood sample processing for flow cytometry analysis was initiated within 1 h from blood collection. Platelet-poor plasma was isolated and frozen at−80°C for future testing within 2 h from the blood draw. Cytology slides were prepared within 1 h of the blood draw.

### Flow cytometry analysis of blood samples

Ethylenediaminetetraacetic acid (EDTA)-anticoagulated blood samples from patients with sICH (100 μl per tube, × 2 replicates) were processed for flow cytometry within 1-h from blood sampling. Flow cytometry buffer comprised of Hank's balanced salt solution plus 2% heat-inactivated fetal bovine serum (FBS) as the blocking agent; staining antibodies: 10 μg/ml of AF-647 labeled hu6g8 mAb, or the corresponding human IgG4-AF647 isotype IgG4, and 2.5 μg/ml anti-CD11b-AF488 or the corresponding mouse IgG1 kappa isotype control, AF-488; staining was performed at 4°C × 30 min with rotation and protected from light. After staining, cells were fixed in 2% phosphate-buffered saline (PBS)-buffered paraformaldehyde (PFA) pH 7.4 at 4 °C, followed by red blood cell (RBC) lysis at room temperature. After the final wash, stained cells were resuspended in 400 μl Hanks' balanced salt solution (HBSS) 2% FBS, filtered, and analyzed on a BD LSR-II flow cytometer. Analysis was performed using FloJo Flow Cytometry Analysis Software (www.FloJo.com). Controls used were both fluorescence minus one (FMO) controls, both isotype controls, and compensation beads for both staining antibodies to check labeled antibody quality.

### Immunofluorescence staining of NET-forming neutrophils

Blood smears were prepared by capillary action from EDTA anticoagulated whole blood (10 μl) samples on a Superfrost Plus Microscope slide (Fisher Scientific, cat# 12-550-15) within 1-h from blood sampling. Blood smears were air-dried for 10 min and then fixed with 100 % methanol (chilled to −20°C) for 10 min. Fixed slides were stored dry in a −20 °C freezer for future immunostaining. Immunofluorescence (IF) staining to detect NET-forming neutrophils was then performed using humanized anti-DEspR IgG4[S228P] hu6g8 labeled in-house with AF568, and commercially available anti-CD11b-AF488, as used for flow cytometry, for direct pair-wise immunostaining; DAPI for DNA detection. Chilled methanol fixation and permeabilization allowed fixation within 1 h from the blood draw. PBS with 5% FBS was used as a blocking and binding solution for primary antibodies.

### Fixed cell imaging of blood smears for quantitation of NET-forming neutrophils

Immunofluorescence imaging was performed as a contract research service at Nikon Imaging Laboratory (Cambridge MA, USA). Slides were imaged using a Nikon Ti2-E Widefield microscope equipped with a Plan Apo λ 20× objective and spectra LED light source and controlled by NIS-Elements. In brief, an automated, JOBS routine in NIS-Elements was used to image 100 evenly spaced positions along an entire slide. At each position, the focus was automatically adjusted with the Perfect Focus System (PFS) and then sequential images with the 395 (blue), 470 (green), and 555 (red) nm LED light sources to detect DAPI (nuclei), Alexa Fluor 488 (CD11b), and Alexa Fluor 568 (DEspR, hu6g8), respectively. Each stack of 100 images was then processed with a General Analysis 3 algorithm in NIS-Elements to segment the nuclei, measure their circularity (Circularity = 4π [area/perimeter^2^], area of a minimum circle enclosing NET-forming neutrophil, perimeter of NET-forming neutrophil with all DNA-extrusions], and quantify the signal intensity of any co-localized CD11b and DEspR expression. Data were exported to a CSV file where the final scoring is completed in Excel.

### Plasma level analysis of biomarkers by ELISA and quantitation of mitochondrial DNA

Individual ELISA protocols were performed as per manufacturer's instructions with the following sample dilutions: for MPO ELISA (Abcam cat# ab195212) plasma dilution 1:1,000; for C5b-9 ELISA (MyBioSource cat# MBS2021557) plasma dilution 1:100; for IL-6 ELISA (Abcam cat# ab46027) plasma dilution 1:2; and for ET-1 ELISA (Abcam cat# ab133030) plasma dilution 1:2.

To compare the levels of mitochondrial to nuclear DNA in human plasma samples, we used the NovaQUANT^™^ Human Mitochondrial to Nuclear DNA Ratio Kit (SIGMA-Aldrich cat# 72620-1KIT) as per the manufacturer's instructions. The kit measures the mtDNA copy number to that of nuclear DNA by Real-Time PCR of specific mitochondrial and nuclear genes optimized for equivalent amplification. Plasma DNA was isolated from 200 μl of plasma using the Quick-cfDNA Serum & Plasma Kit (Zymo Research, cat# D4076) as per the manufacturer's instruction.

### Statistical analysis

For this pilot observational study, correlations were calculated using the Spearman Rank Order correlation test (GraphPad PRISM version 9.3), and power calculations were determined using the SigmaPlot 11.0 software. Bonferroni correction for *p*-values was performed for multiple comparisons. All data sets conformed to the assumptions of each specific statistical test. *p* < 0.05 was considered statistically significant, sufficient power 0.8. For group comparisons of rank, we used the two-tailed unpaired Mann–Whitney rank test (GraphPad PRISM version 9.3). Hedge's g corrected effect size was used with < 4% correction for sample size < 20.

## Results

### Descriptive analysis

A total of 13 patients were prospectively enrolled: age range 32–83 years, six (46%) women, two with no medical history of hypertension and were normotensive on arrival, and one had an unknown medical history. The ICH score ranged from 0 to 4. Eleven patients (85%) had intraventricular hemorrhage (IVH) with a modified Graeb score ranging from 0 to 21; six patients received an external ventricular drain for hydrocephalus. The ICU length of stay (LOS) ranged from 4 to 39 days. Four (2 female) patients with sICH (31%) were subsequently placed on comfort measures only (CMO) on days 4, 6, 7, and 16 and died on average 1.75 days after CMO designation. Among non-survivors, 2 subjects had lobar (from a total of 3 with lobar location) and 2 had thalamic hemorrhages (from a total of 9 with thalamic or basal ganglia hemorrhages). Among surviving patients, 4/9 (44%) were women and 7/9 (78%) had a history of hypertension. Demographic data are summarized in Table 1 and detailed in Supplementary Table S1 with clinical and radiological measures of sICH severity used for analyses.

**Table 1:** Summary of key patient data.

										**Obtained on day of FCM** ± **1-day**							
***ID #***	***Sex***	***Age*** ***(y)***	***PMHx***	***ICH*** ***site***	***# ICU*** ***days***	***CMO*** ***(day)***	***Death*** ***(day)***	***ICH*** ***score***	***90d*** ***mRS***	***FCM*** ***(days)***	***GCS***	***IPH*** ***vol*** ***(ml)***	***PHE*** ***vol*** ***(ml)***	***mGS***	***NLR***	***#DEspR***+***CD11b***+	
																**Ns/**μ**L**	**NET**+ **Ns/**μ**L**
# 1	F	71	HT	Thalamus	39	N/A	N/A	2	5	4	7	5.52	14.07	13	5.7	2571	640
# 2	M	67	unk	Thalamus	16	16	16	2	6	4	12	13.87	13.92	9	17.3	4017	663
# 3	F	82	HT	Thalamus	7	7	7	3	6	2	13	17.82	12.75	13	5.6	2470	822
# 4	M	60	HT	Thalamus	6	N/A	N/A	1	3	3	15	0.71	3.78	12	4.2	285	89
# 5	M	71	HT	Cor. radiata	7	4	5	3	6	2	7	1.57	4.86	21	10.1	5950	3343
# 6	F	83	NT	Occ. Par.	11	6	11	4	6	5	10	44.70	48.40	1	5.6	5072	4645
# 7	M	51	HT	Occ. Par.	6	N/A	N/A	0	2	4	15	0.77	1.93	0	1.5	909	519
# 8	F	60	HT	Thalamus	19	N/A	N/A	2	5	8	9	6.09	11.91	4	3.0	2140	1252
# 9	F	83	NT	Thalamus	2	N/A	N/A	1	3	2	14	2.92	0.77	0	1.4	950	651
#10	M	63	HT	Thalamus	10	N/A	N/A	2	5	0	6	25.20	19.90	13	8.6	4590	1260
#11	F	71	NT	Basal Ganglia	2	N/A	N/A	1	3	1	14	0.63	2.47	12	1.6	1050	573
#12	M	32	HT	Thalamus	39	N/A	N/A	2	3	4	12	1.89	1.89	9	2.7	282	213
#13	M	37	HT	Basal Ganglia	4	N/A	N/A	0	0	3	14	12.60	8.40	1	1.9	935	655
										Avg 3.2							

CMO, comfort measures only; cor. Radiata, corona radiata; FCM, flow cytometry; GCS, Glasgow Coma Scale; HT, hypertensive; ICH score, intracerebral hemorrhage score; #ICU, number of days in intensive care unit; IPH vol, intraparenchymal hemorrhage volume; mGS, modified Graeb score for intraventricular hemorrhage; mRS, modified Rankin Scale; N/A, not applicable; Ns, neutrophil counts per microliter whole blood; NET+ Ns/μl, circulating NET-forming neutrophils per microliter whole blood detected on immunofluorescence blood smear analysis; NLR, neutrophil-lymphocyte ratio; NT, normotensive; Occ. Par., occipital parietal; PHE vol, perihematomal edema volume; PMHx, past medical history for hypertension; unk, unknown.

### Validation of *ex vivo* neutrophil subset analysis

**Figure 1:**
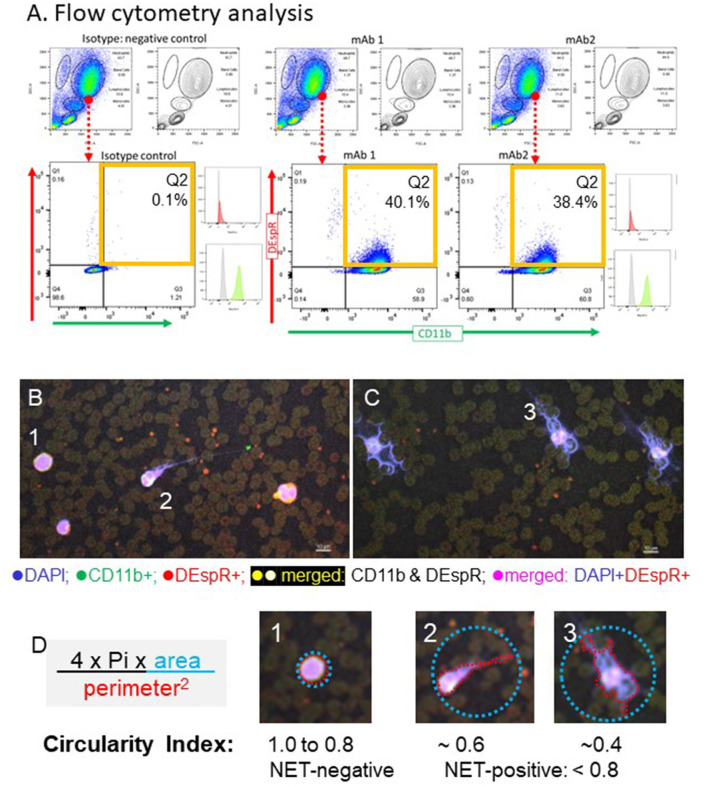
Representative images of identification of DEspR+CD11b+ “rogue” neutrophils by flow cytometry and neutrophil extracellular trap (NET)-forming neutrophils by immuno-cytology in patients with spontaneous intracerebral hemorrhage (sICH) whole blood. **(A)** Representative flow cytometry (FCM) analysis, done in duplicates, of DEspR+CD11b+ neutrophils (Ns) of the first sICH consented subject. Top row: FSC, forward side scatter (size), SSC, side scatter (granularity), → encircled neutrophil cloud identified on a dot plot, corroborated on contour plot, and distinguished from monocytes and lymphocytes. Isotype controls for both antibody probes vs. double immunotyping with anti-DEspR (hu6g8)-AF647 and anti-CD11b-AF488. □ Quadrant 2 (Q2) for DEspR+CD11b+ neutrophils. Mean fluorescence intensity (MFI) histograms shown: isotype ■, hu6g8 ■, and CD11b ■. **(B)** Representative images of immunofluorescence (IF)-stained peripheral blood smears from a patient survivor with sICH and **(C)** non-survivor on day of flow cytometry analysis. DEspR+CD11b+ NET-forming neutrophils with still intact cell membranes are noted in both. NET-forming neutrophil extruded DNA wrapped around red blood cells (RBCs) noted predominantly in sICH non-survivors. Bar = 10 μm. **(D)** Quantitation of intravascular NET-formation using shape analysis circularity index formula: 4 ^*^ Pi ^*^ area of the smallest circle that encompasses the whole NET-forming neutrophil divided by perimeter-squared of the traced outline of the DAPI-stained DNA extruded from the neutrophil with the still intact cell membrane. Panels #1: example of non-NET-forming neutrophil with circularity index 1.0; #2: NET-forming neutrophil with circularity index ~0.6; #3: NET-forming neutrophil with circularity index ~ 0.4. For quantitation: NET-negative: circularity index 0.8 to 1.0; NET-positive: circularity index < 0.8. Automated imaging and quantitation performed.

We validated our *ex vivo* analysis protocols of neutrophil subsets in patients with sICH guided by our prior analysis of neutrophil subsets in patients with ARDS (12). We determined optimal conditions for *ex vivo* analysis by flow cytometry and immunofluorescence cytology. We ascertained duplicate reads on flow cytometry and gated-out background and non-specific binding detected in isotype controls in flow cytometry (Figure 1A) and ascertained target-specific immune-fluorescence staining for automated quantitation of NET-forming neutrophils by circularity index (Figures 1B–D). To validate comparative correlation analysis of flow cytometry, immunofluorescence-cytology, and plasma biomarker analysis data, we used the same blood sample for each patient for all assays and the identical protocol across all patient samples. The NLR was derived from the same-day CBC-differential, with the exception of 1 subject's CBC-differential procured the day before flow cytometry.

To eliminate confounders arising from the dynamic day-to-day changes after the onset of sICH, we obtained CT scan-based radiological measurements for IPH volumes, PHE volumes, midline shift, and modified Graeb score (mGS) for IVH on CTs performed on the day of flow cytometry, ± 1 day (Supplementary Table S1). There was only one exception: one patient with sICH with an ICH score of 0 only had an admission CT, 3 days prior to the day of flow cytometry analysis. CT-based measures were ascertained by > 0.9 correlation of blinded measurements by two independent board-certified neurointensivists (CT and DG) and by computerized measurement.

In total, we validated 21 parameters for correlation matrix analysis to assess the role of DEspR+CD11b+ neutrophil subsets: 8 flow cytometry measures of DEspR+ leukocyte-subsets, 5 plasma biomarkers relevant to neutrophil-mediated secondary brain injury in sICH as comparators, and 8 clinical and radiological parameters of sICH severity for correlation (Supplementary Table S1). With the exception of admission ICH score and 90-day mRS, the median/average day of same-day parameters was 3 days.

### Correlation matrix analysis

To test the hypothesis that DEspR+CD11b+ neutrophils are associated with neutrophil-mediated brain injury in sICH, we performed a Spearman rank correlation matrix analysis of validated data (Supplementary Figure S1, Supplementary Table S2) and observed the following.

First, correlation analysis of measures of primary injury (IPH volume) and secondary brain injury (PHE volume) obtained on the day of FCM analysis [t1] shows a strong significant correlation (*r* = 0.78, *p*^*B*^ = 0.004) with each other and moderate correlation with midline shift, but not with mGS for IVH (Table 2, Supplementary Table S2). PHE volumes exhibited a significant correlation with clinical measures of sICH severity (admission ICH score, same-day GCS score, and 90-day mRS), whereas IPH volumes did not in this pilot study (Table 2).

**Table 2:** Spearman rank correlation coefficients (*r)* among clinical and radiological measures of sICH severity (*n* = 13).

	**Admission** **ICH–Score**	**t**−**1** **GCS–Score**	**90–day** **mRS**	**t**−**1** **IPH–vol**	**t**−**1** **PHE–vol**	**t**−**1** **Mid–shift**	**t**−**1** **mGS**
	***r* [*p*^B^]**	***r* [*p*^B^]**	***r* [*p*^B^]**	***r* [*p*^B^]**	***r* [*p*^B^]**	***r* [*p*^B^]**	***r* [*p*^B^]**
**t−1 IPH–vol**	0.54 [n.s.]	−0.51 [n.s.]	0.53 [n.s.]		0.78 [0.004^B^]	0.67 [0.030^B^]	−0.01 [n.s.]
**t−1 PHE–vol**	0.59 [n.s.]	−0.64 [0.042^B^]	0.65 [0.036^B^]	0.78 [0.004^B^]		0.68 [0.026^B^]	0.35 [n.s.]

Pilot observational study (single site) n = 13 subjects. Spearman rank order correlation, with Bonferroni corrected p–value, p^B^.

t−1, day of flow cytometry analysis; GCS–score, Glasgow Coma Scale score; ICH Score, intracerebral hemorrhage score; IPH–vol, intraparenchymal hemorrhage volume (ml); mGS, modified Graeb scale for intraventricular hemorrhage; Mid–shift, midline shift, (mm); mRS, modified Rankin Scale; PHE–vol, perihematomal volume (ml).

r, Spearman correlation coefficient rho; rho ≥ 0.71: α < 0.05, power > 0.8 with n = 13 (bold). Significant (0 < 0.05) correlation rho < 0.71 but power < 0.08. n.s., not significant.

**Figure 2:**
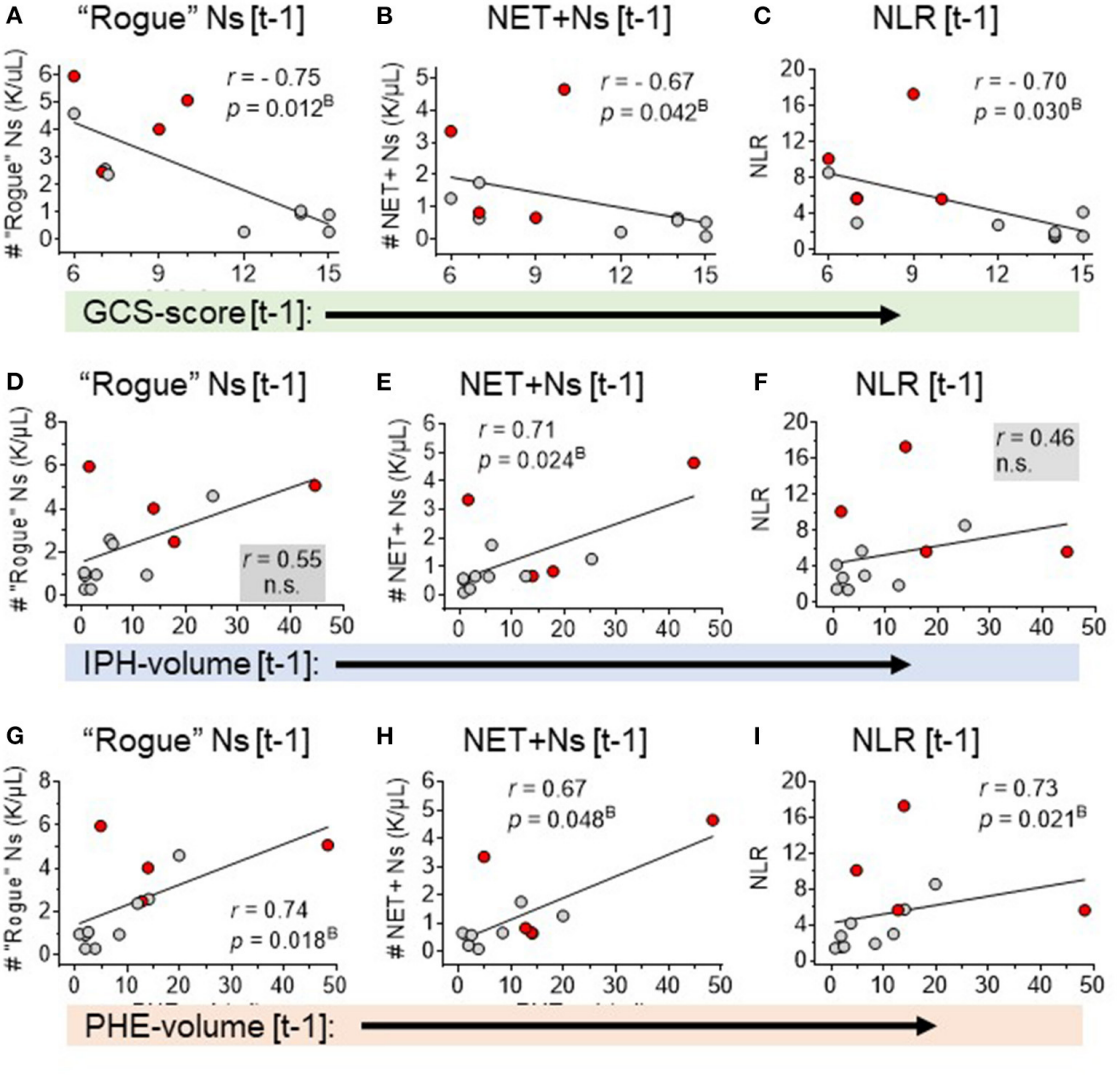
Spearman rank correlation analyses of three neutrophil-based markers with sICH severity: GCS-score, IPH-volume, and PHE-volume. Correlation of GCS-score [t-1] with **(A)** DEspR+CD11b+ neutrophil counts, K/μl (“rogue” Ns [t-1]). **(B)** NET-forming neutrophil counts, K/μl, (NET+Ns [t-1] and **(C)** neutrophil lymphocyte ratio (NLR). Correlation of IPH volume [t-1] with: **(D)** DEspR+CD11b+ neutrophil counts, K/μl (“rogue” Ns [t-1]), **(E)** NET-forming neutrophil counts, K/μl (NET+Ns [t-1], and **(F)** neutrophil-lymphocyte ratio (NLR). Correlation of PHE-volume [t-1] with **(G)** DEspR+CD11b+ neutrophil counts, K/μl (“rogue” Ns [t-1]), **(H)** NET-forming neutrophil counts, K/μl (NET+Ns [t-1]), and **(I)** neutrophil-lymphocyte ratio (NLR). All measurements were obtained on the same day ± 1-day [t-1] as the day of flow cytometry analysis; red • symbols represent sICH-subject nonsurvivors; gray • symbols mark values for sICH-subject survivors; *r*, Spearman rank correlation coefficient; *p*^B^, *p*-value with Bonferroni correction. ns, not significant.

**Figure 3:**
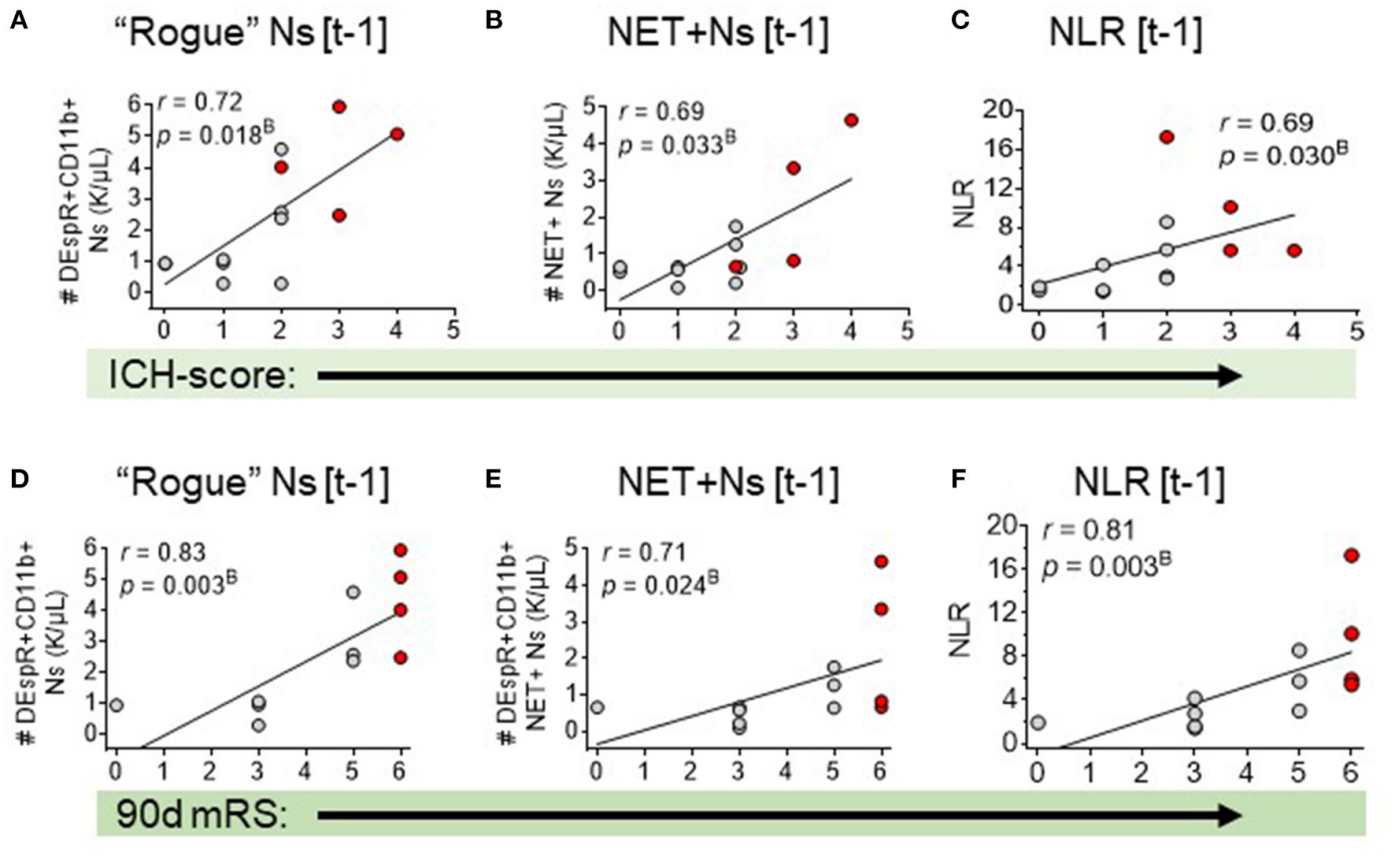
Spearman rank correlation analysis of three neutrophil-based markers and sICH outcomes: admission ICH score and 90-day mRS. Correlation of admission ICH score with: **(A)** DEspR+CD11b+ neutrophil counts, K/μl, (“rogue” Ns [t-1]), **(B)** NET-forming neutrophil counts, K/μl, (NET+Ns [t-1], and **(C)** neutrophil-lymphocyte ratio (NLR). Correlation of 90-day mRS with: **(D)** DEspR+CD11b+ neutrophil counts (“rogue” Ns [t-1]), **(E)** NET-forming Ns (NET+Ns [t-1], **(F)** neutrophil lymphocyte ratio (NLR). [t-1], on day of flow cytometry; Red • symbols represent sICH-subject non-survivors; gray • symbols mark values for sICH-subject survivors; *r*, Spearman rank correlation coefficient; and *p*^B^, *p*-value with Bonferroni correction.

Second, correlation analysis of DEspR±CD11b± leukocyte subsets (neutrophils, monocytes, and lymphocytes) identified by flow cytometry (Supplementary Table S2), Supplementary Figure S1) differentiated three same-day neutrophil markers with highest correlation coefficients (*r* > 0.7, power > 0.8, significant Bonferroni corrected *p*-value) with sICH severity measures: (a) DEspR+CD11b+ neutrophil (N) counts, (b) DEspR+CD11b+ NET-forming neutrophil counts (NET+N counts), and (c) NLR. Among these 3 top neutrophil markers, the DEspR+CD11b+ N counts exhibited strong correlations with multiple sICH measures of clinical severity: GCS score, ICH score, 90-day mRS, and radiological measures of secondary brain injury (PHE volume) measured on the same ± 1 day as flow cytometry (FCM) determination of DEspR+CD11b+ neutrophil counts (Figures 2, 3, Table 3). DEspR+CD11b+ NET+ N counts exhibited a significantly strong correlation with GCS score (Figure 2), 90-day mRS (Figure 3), and IPH volume (Figure 2). Moderate Spearman rank correlations (*r* > 0.66, Bonferroni corrected *p*-value < 0.05, but power < 0.8) were observed between DEspR+ NET+Ns with same-day GCS score, PHE-vol (Figure 2), and admission ICH score (Figure 3, Table 3). Concordantly, same-day NLR exhibited significant strong correlations with PHE volume and 90-day mRS and moderate correlation with same-day GCS score and admission ICH score (Figures 2, 3, Table 3).

**Table 3:** Spearman rank correlation analysis of top 3 peripheral neutrophil markers with clinical and radiological measures of sICH severity (*n* = 13).

	***Clinical Measures***			***Radiological PARAMETERS***	
	***t**−**1 GCS Score***	***Admission ICH Score***	***90d mRS***	***t**−**1*** ***IPH–vol***	***t***−***1 PHE–vol***
***MARKER***	***r* [*p^***B***^*]**	***r* [*p^***B***^*]**	***r* [*p^***B***^*]**	***r* [*p^***B***^*]**	***r* [*p^***B***^*]**
**t−1 # DEspR+CD11b+ Ns (K/μL)**	**−0.75 [0.012^**B**^]**	**0.72 [0.018^**B**^]**	**0.83 [0.003^**B**^]**	0.55 [n.s.]	**0.74 [0.018^**B**^]**
**t−1 # DEspR+CD11b+NET+ Ns (K/μL)**	−0.67 [0.042^B^]	0.69 [0.033^B^]	**0.71 [0.024^**B**^]**	**0.71 [0.024 ^**B**^]**	0.67 [0.048^B^]
**t−1 Neutrophil–to–Lymphocyte Ratio (NLR)**	−0.70 [0.030^B^]	0.69 [0.030^B^]	**0.81 [0.003^**B**^]**	0.46 [n.s.]	**0.73 [0.021^**B**^]**

Pilot observational study (single site) n = 13 subjects. Spearman rank order correlation coefficient (r), with Bonferroni corrected p–value × 3, p^B^.

t−1, day of flow cytometry analysis; # DEspR+CD11b+ Ns: total number (#) in K/μl of DespR+C11b+ neutrophils (Ns); # DespR+CD11b+NETosing Ns: number of DespR+CD11b+ NET–forming Ns (% DespR+CD11b+ NET–forming Ns × total number of DespR+CD11b+ Ns); NLR, absolute neutrophil count/absolute lymphocyte ratio; GCS Score, Glasgow Coma Scale; ICH Score, admission intracerebral hemorrhage score; 90–day mRS, 90–day modified Rankin Scale score; IPH–vol, intraparenchymal volume (ml); PHE–vol, perihematomal edema volume (ml).

r, Spearman correlation coefficient rho; r ≥ 0.71: α < 0.05, power > 0.8 with n = 13 (bold). Significant (p < 0.05) correlation, Significant (p < 0.05) correlation rho < 0.71, but power < 0.8.

### Comparative mapping of the top 3 neutrophil-based parameters

**Figure 4:**
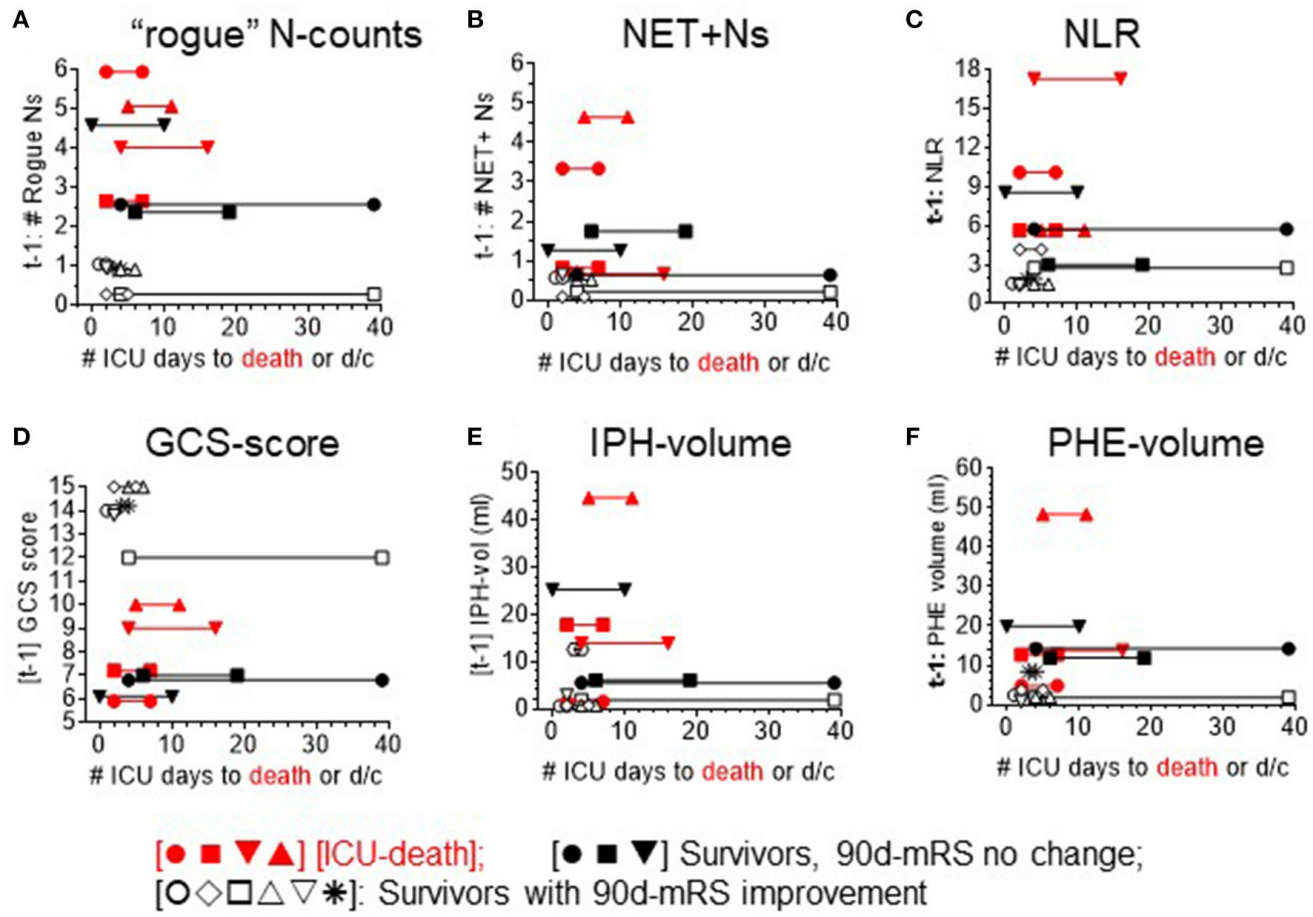
Mapping of each sICH patient's ICU course (X-axis) per level of different analysis parameters. ICU course: timeline to discharge (d/c) or death plotted against level of neutrophil measures: **(A)** DEspR+CD11b+ neutrophil counts (“rogue” N counts, K/μl). **(B)** intravascular NET+ neutrophils (NET+N counts, K/μl). **(C)** neutrophil-to-lymphocyte ratio (NLR), clinical measure of neurological deficits: **(D)** Glasgow Coma Scale-score (GCS), as well as radiological measures of sICH severity: **(E)** intraparenchymal hemorrhage (IPH)-volume, and **(F)** perihematomal edema (PHE)-volume. Red symbols [•■▾▴] ICU-death [nonsurvivors]; black symbols: ICU-discharge (d/c) [survivors]; solid black [•■▾]: no improvement in 90-day-mRS (non-imp); and open black symbols [○♢□▵▿*]: 90-day mRS improved (imp).

To advance insight into the clinical relevance of correlations detected, we mapped the top 3 neutrophil-based parameters (DEspR+CD11b+ N counts, NET+ N counts, and NLR) that exhibited the strongest correlations with clinical and radiological measures of sICH severity, along the timeline of the number of days in the ICU until ICU death or discharge (Figures 4A–C). This mapping shows pattern differences between non-survivors and survivors. Parallel mapping of GCS scores, IPH volume, and PHE volume as current standard-of-care comparators (Figures 4D–F) shows similarities with the timeline maps of neutrophil-based markers. Comparative mapping shows overlaps between survivors and non-survivors among neutrophil-based markers and clinical standard measures: GCS and IPH- and PHE-volumes (Figures 4A–F) delineating a potential clinical subgroup.

### Analysis of the DEspR+CD11b+ neutrophil subset in survivors

**Figure 5:**
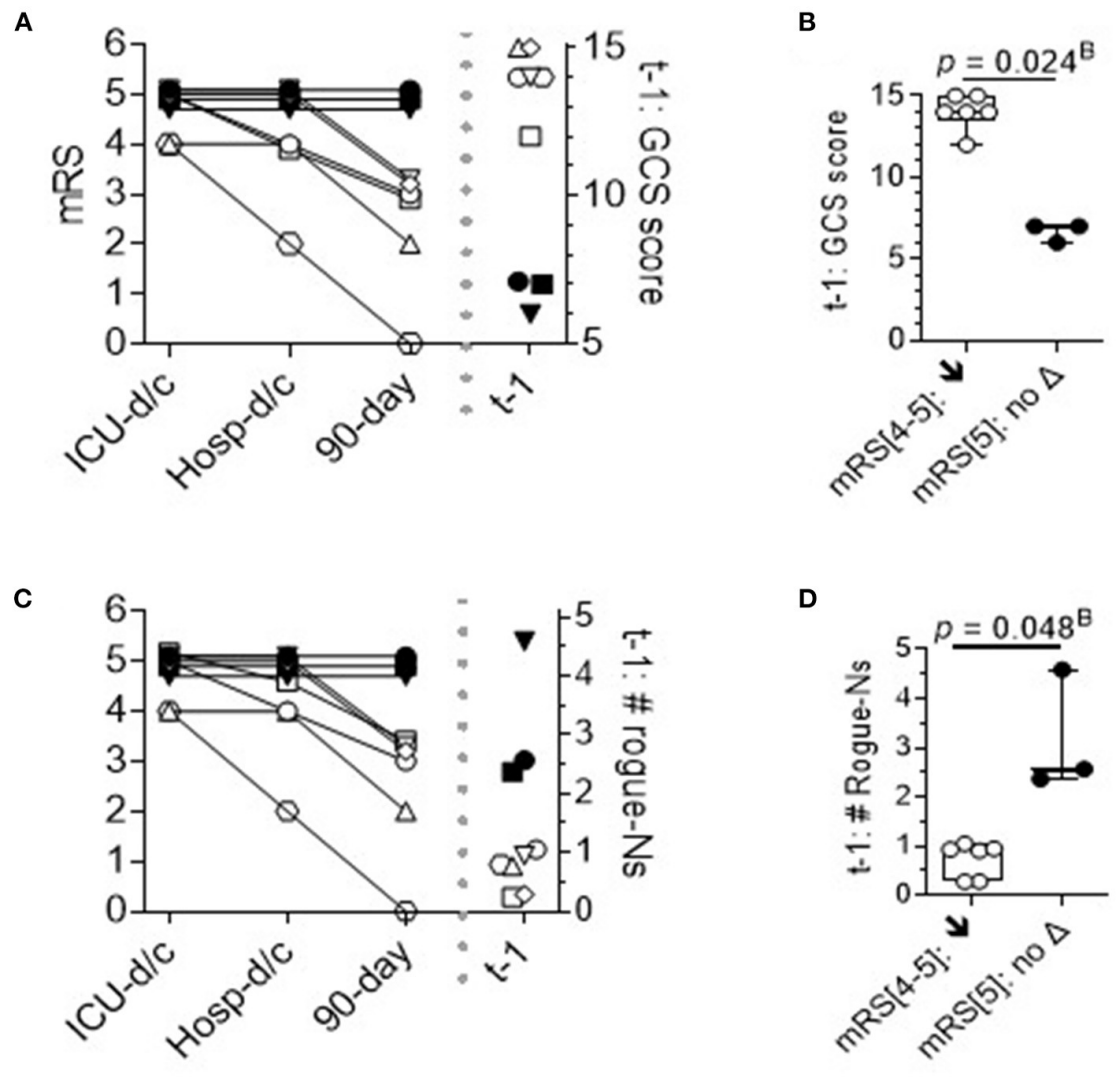
Time course of modified Rankin Scale (mRS) scores in sICH survivors. Per patient time course of mRS at discharge from the ICU, discharge from the hospital, and 90 days from sICH diagnosis: survivors (*n* = 9), stratified by no improvement (▾■•) vs. 90-day improvement in mRS (*n* = 6: ○△▽□♢^*^). **(A)** Per patient mRS plot and corresponding GCS scores on the day of flow cytometry analysis. Stratification of survivors with improvement (**↓**) vs. without improvement (no Δ) in mRS scores. **(B)** t-1 GCS scores: 2-tailed Mann-Whitney rank test *p* = 0.024^B^ with Bonferroni correction, Hedge's g effect size: 7.3 less 4% correction. **(C)** Per patient mRS plot and corresponding levels of DEspR+CD11b+ neutrophil counts. **(D)** t-1 #DEspR+CD11b+ neutrophil counts: two-tailed Mann-Whitney rank test, *p* = 0.048^B^ with Bonferroni correction, Hedge's g effect size 3.3 less 4% correction. ICU-d/c; mRS at discharge from the ICU; Hosp-d/c, mRS at discharge from the hospital; 90-day, mRS at 90 days from sICH admission; t-1, day of flow cytometry; mRS[4–5] **↓**; patients with sICH with t1 mRS[4–5] that exhibited a decrease in mRS score or improved; and mRS[5]: no Δ, no change in mRS score.

To further analyze the DEspR+CD11b+ neutrophil subset among sICH survivors, we mapped the temporal course of the mRS scores at three time points: at ICU discharge, hospital discharge, and at 90 days from sICH symptoms. The trend map of mRS scores was then compared with the corresponding per-patient GCS scores obtained on the day of flow cytometry (t-1) (Figures 5A,B) and corresponding per-patient levels of t-1 DEspR+CD11b+ neutrophil counts (Figures 5C,D). This trend comparison shows the alignment of non-improvement in mRS scores with higher rogue DEspR+CD11b+ neutrophil counts and with worse (lower) GCS scores. Mann-Whitney rank sum analysis detected significant differences in sICH survivors with and without improvement in mRS scores for both GCS scores (Figure 5B) and DEspR+CD11b+ neutrophil counts (Figure 5D).

### Emerging interrelationships among neutrophil-based measures, NLR, and index ICH score

**Figure 6:**
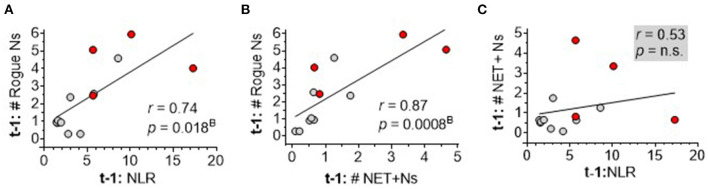
Spearman rank correlation analysis of three neutrophil-based markers among each other. **(A)** Correlation of same day (t-1) DEspR+CD11b+ neutrophil counts, (Rogue Ns, K/μl), (t-1: # Rogue Ns) with neutrophil-lymphocyte ratio (NLR); **(B)** correlation of t-1:# Rogue Ns with NET-forming neutrophil counts (NET+Ns, K/μl), (t1: # NET+Ns); and **(C)** correlation of t-1: # NET+Ns with NLR. Ns, neutrophils; t1: day of flow cytometry; red • symbols represent sICH nonsurvivors; gray • symbols mark values for sICH survivors; *r*, Spearman rank correlation coefficient; *p*^B^, *p*-value with Bonferroni correction. n.s. not significant.

To dissect the interrelationships among the top three neutrophil measures correlating with sICH severity, inter-marker Spearman rank correlation analysis showed a significant correlation between DEspR+CD11b+ neutrophil counts and NLR (Figure 6A) and between DEspR+CD11b+ neutrophil counts and NET+ N counts (Figure 6B). In contrast, NLR and DEspR+CD11b+ NET+ N counts did not exhibit a significant correlation with each other (Figure 6C).

To explore the interrelationship of neutrophil measures with the clinical index ICH-score, we performed comparative Spearman correlation analyses of the index ICH-score alone, as well as 2-marker and 4-marker combinations with the index ICH-score. We detected a very strong correlation (*r* = 0.92) for the index ICH-score, and 2-marker combinations, with 90-day mRS (Table 4). Even stronger correlation was observed (*r* = 0.94, *p*^*B*^ = 10^−5^) for the 4-marker combination: sum of the index ICH-score, DEspR+ N counts, DEspR+ NET+ N counts, and NLR (Table 4).

**Table 4:** Spearman rank correlation: combinatorial analysis sICH cohort (*n* = 13).

	**90d–mRS**	
**Markers**	***r***	***p**^*B*^* **value**
ICH Score	0.917	1.1x10^−4^
ICH Score + NLR	0.901	4x10^−4^
ICH Score + Ns	0.921	1.5x10^−4^
ICH Score + [NET+Ns]	0.921	1.5x10^−4^
ICH Score + NLR + Ns + [NET+Ns]	**0.941**	4.5x10^−5^

NLR, neutrophil lymphocyte ratio calculated from ratio of absolute neutrophil to absolute lymphocyte counts obtained from hospital complete blood count (CBC)–differential; ICH, intracerebral Hemorrhage; Ns, DEspR+CD11b+ neutrophil counts (K/μl); NET+Ns, DEspR+CD11b+NET+ neutrophil counts (K/μl).

Spearman rank order correlation analysis. r, Spearman correlation coefficient; for α < 0.05, power > 0.8 with n = 13, r > 0.710. Bonferroni corrected p–values (p^B^).

### Analysis of the intravascular proinflammatory milieu in sICH

As pathophysiological comparators, we tested four biomarkers known to be elevated in sICH: interleukin-6 (IL-6) as a marker of monocyte crosstalk with IL-6 receptor-positive neutrophils (15), soluble terminal complement complex (sC5b-9) as a peripheral marker for complement activation (16), myeloperoxidase (MPO) as a marker for neutrophil activation (17), and endothelin-1 (ET-1), reported to be increased in sICH (18), enhance neutrophil activation (19) and an activating ligand for DEspR (20). We also analyzed the plasma mitochondrial DNA/nuclear DNA (mt/nDNA) ratio, expected to be elevated with increased intravascular NET+N counts (21).

Interleukin-6, sC5b-9, ET-1, and mt/nDNA ratio were elevated above normal levels but not MPO (Supplementary Table S1). Spearman rank correlation matrix analysis did not detect significant correlation coefficients with sICH severity measures. A moderate correlation was detected (*r* = 0.5) for IL-6 with GCS score, sC5b-9 with ICH score and 90-day mRS, and mt/nDNA ratio with GCS score and 90-day mRS (Supplementary Figure S1, Supplementary Table S3).

## Discussion

The detection of circulating DEspR+CD11b+ rogue neutrophil subset and NET-forming neutrophils at acute sICH (average 3.3 days from ICU admission) demonstrates the clinical feasibility of tracking neutrophil subsets and phenotypes in sICH for translational research. The observed correlations of three different peripheral neutrophil biomarkers—DEspR+CD11b+ N counts and NET+N counts, and NLR with 90-day mRS—collectively support the role of neutrophil-mediated secondary brain injury as a determinant of sICH outcome. These correlations suggest that peripheral neutrophils reflect ongoing neutrophil-mediated secondary brain injury in sICH, a notion supported by RNA-profiling data showing that peripheral neutrophils exhibit similar RNA profiles as brain neutrophil infiltrates in patients with sICH at 72 h (8). Furthermore, as our neutrophil measures were obtained on average on day 3, observed correlations with subsequent 90-day mRS suggest putative consequential role(s).

The correlation of same day (~3 days) DEspR+CD11b+ N counts and NLR with PHE volume aligns with the reported association of PHE volume 72 h after acute sICH with mortality (22). As PHE is a radiological marker of secondary brain injury (2), the correlation of DEspR+CD11b+ N counts and NLR with PHE volume but not with IPH volume, the primary injury, is concordant with the differential pathogenesis of PHE and IPH volume expansion. These observations align with clinical trial findings showing that hematoma evacuation lowers mortality without functional improvement (3) and support the recommendation that secondary brain injury at acute ICH needs to be addressed (23).

In contrast to DEspR+CD11b+ neutrophil counts and NLR which did not correlate with IPH volume, the differential stronger correlation of intravascular NET+ neutrophil counts with IPH volume compared with PHE volume suggests new insight. Increased day 3 intravascular NET+ neutrophil counts are concordant with the detection of increased NETs in the hematoma and perihematomal areas in postmortem brain sections obtained from patients with sICH who died 72 h after sICH (7). The dual observation of increased intravascular NET+ neutrophil counts and increased plasma sC5b-9 biomarker levels of complement activation suggests self-sustaining reciprocal interactions, as complement activation induces NET-formation, and vice versa (24). Additionally, as complement activation in the brain occurs on entry of blood into the brain (16, 25), complement-mediated recruitment of activated neutrophils and NET-formation likely occurs from sICH onset independent of microglial activation.

The correlation of DEspR+CD11b+ neutrophil counts with NLR in patients with sICH suggests that the DEspR+ neutrophil subset contributes to increased NLR in sICH given observed delayed apoptosis in *ex vivo* analysis of DEspR+CD11b+ neutrophils (12). The correlation of DEspR+CD11b+ neutrophil counts with NET+ neutrophil counts indicates a predisposition of DEspR+ neutrophils to intravascular NET formation. Based on known neutrophil- and NET-mediated cell injury mechanisms (26), DEspR+CD11b+ rogue neutrophils prone to NET-formation could provide injury mechanisms that contribute to the association of high NLR with worse in-hospital mortality (27), worse 90-day outcomes in sICH (28, 29), and the association of high postoperative NLR with mortality after hematoma evacuation (30). These relational observations are represented in the very strong correlation (*r* > 0.94) detected for the 4-marker combination stronger than the ICH score or any neutrophil marker alone in this study and consistent with an earlier report of a stronger association of combined NLR and modified ICH score with 30-day mRS (31, 32). These concordant observations reinforce neutrophil-mediated secondary brain injury as a key determinant of outcomes in sICH and the need for neutrophil subset-specific therapeutic approaches, given shortfalls of nonspecific anti-inflammatory glucocorticoid and edema-reducing hyperosmolar therapy (33).

Finally, this pilot study shows the importance of *ex vivo* analysis of patients with sICH circulating neutrophils and NET-forming neutrophils to advance the study of neutrophil-mediated secondary tissue injury in sICH. This is particularly relevant to the study of neutrophils given known species-specific molecular and functional differences between human and mouse model neutrophils (34) and the accessibility of peripheral neutrophils for *ex vivo* analyses. These considerations validate *ex vivo* analysis as an important translational milestone able to attain early verification of potential therapeutic targets in human sICH, as recommended by HEADS (35) in order to address historically poor translatability of preclinical studies in sICH (1).

### Limitations of this study

This prospective pilot observational study is limited by being from a single center and a relatively small number of subjects. This pilot study is also limited by logistic limitations determining the cohort of consented subjects only and varied times of earliest possible blood sampling, which were dependent on the timing of completed consent and timing to allow flow cytometry analysis within 1 h from blood sampling to eliminate neutrophil activation confounders that can occur when beyond 1 h.

## Conclusion

Our data identify DEspR+CD11b+ neutrophils prone to intravascular NET formation as a putative “rogue” neutrophil subset implicated in secondary brain injury in sICH. More importantly, the convergence of our *ex vivo* data with cumulative reports implicating neutrophils, along with the potential for targeted inhibition of DEspR+CD11b+ neutrophils shown in patient samples *ex vivo* with ARDS (12) and preclinical efficacy of DEspR-inhibition observed in a spontaneous ICH rat model (14), altogether provide the basis to further test DEspR+CD11b+ neutrophils and NET-forming neutrophils as a potential actionable neutrophil-specific therapeutic target in sICH.

## Data availability statement

The original contributions presented in the study are included in the article/Supplementary material, further inquiries can be directed to the corresponding author.

## Ethics statement

The studies involving human participants were reviewed and approved by Institutional Review Board, Boston University School of Medicine. The patients/participants provided their written informed consent to participate in this study.

## Author contributions

Design and overall supervision of this study: VH, DG, and NR-O. Screening, consent, and sample procurement: RD. General Clinical Research Unit staff, analysis of patient samples by flow cytometry, immunofluorescence staining, and ELISA: VH, MN, JM, and NR-O. Clinical data measurements: CT and DG. Manuscript writing: VH, CT, DG, and NR-O. Manuscript review: All authors. All authors contributed to the article and approved the submitted version.

## Funding

This study was funded by a sponsored research grant from NControl Therapeutics, Inc. to Boston University School of Medicine: principal investigator: NR-O; coinvestigators: DG and VH. This study was supported by the BUSM-CTSI GCRU [NIH/NCATS 1UL1TR001430].

## Conflict of interest

Boston University holds awarded and pending patents on DEspR. VH and NR-O are co-inventors filed by Boston University. These patents comprise the option granted for exclusive license to NControl Therapeutics, Inc. VH and NR-O are scientific co-founders of NControl Therapeutics, and paid consultants with equity in NControl Therapeutics, Inc. NControl Therapeutics was not involved in the design, conception, data interpretation, or manuscript preparation. The remaining authors declare that the research was conducted in the absence of any commercial or financial relationships that could be construed as a potential conflict of interest.

## Publisher's note

All claims expressed in this article are solely those of the authors and do not necessarily represent those of their affiliated organizations, or those of the publisher, the editors and the reviewers. Any product that may be evaluated in this article, or claim that may be made by its manufacturer, is not guaranteed or endorsed by the publisher.

## References

[1] Kearns KN Ironside N Park MS Worrall BB Southerland AM Chen CJ. Neuroprotective therapies for spontaneous intracerebral hemorrhage. Neurocrit Care. (2021) 35:862–86. 10.1007/s12028-021-01311-3 34341912

[2] Selim M Norton C. Perihematomal edema: implications for intracerebral hemorrhage research and therapeutic advances. J Neurosci Res. (2020) 98:212–18. 10.1002/jnr.24372 30575082 PMC6588515

[3] Hanley DF Thompson RE Rosenblum M Yenokyan G Lane K McBee N. Efficacy and safety of minimally invasive surgery with thrombolysis in intracerebral haemorrhage evacuation (MISTIE III): a randomised, controlled, open-label, blinded endpoint phase 3 trial. Lancet. (2019) 393:1021–32. 30739747 10.1016/S0140-6736(19)30195-3 PMC6894906

[4] Aronowski J Zhao X. Molecular pathophysiology of cerebral hemorrhage: secondary brain injury. Stroke. (2011) 42:1781–86. 10.1161/STROKEAHA.110.596718 21527759 PMC3123894

[5] Qin J Li Z Gong G Li H Chen L Song B. Early increased neutrophil-to-lymphocyte ratio is associated with poor 3-month outcomes in spontaneous intracerebral hemorrhage. PloS ONE. (2019) 14:e0211833. 10.1371/journal.pone.0211833 30730945 PMC6366889

[6] Shtaya A Bridges LR Esiri MM Lam-Wong J Nicoll JAR Boche D. Rapid neuroinflammatory changes in human acute intracerebral hemorrhage. Ann Clin Transl Neurol. (2019) 6:1465–79. 10.1002/acn3.50842 31402627 PMC6689697

[7] Puy L Corseaux D Perbet R Deramecourt V Cordonnier C Bérézowski V. Neutrophil extracellular traps (NETs) infiltrate haematoma and surrounding brain tissue after intracerebral haemorrhage: a post-mortem study. Neuropathol Appl Neurobiol. (2021) 47:867–77. 10.1111/nan.12733 33971034

[8] Durocher M Ander BP Jickling G Hamade F Hull H Knepp B. Inflammatory, regulatory, and autophagy co-expression modules and hub genes underlie the peripheral immune response to human intracerebral hemorrhage. J Neuroinflammation. (2019) 16:56. 10.1186/s12974-019-1433-4 30836997 PMC6399982

[9] Liu S Liu X Chen S Xiao Y Zhuang W. Neutrophil-lymphocyte ratio predicts the outcome of intracerebral hemorrhage: a meta-analysis. Medicine (Baltimore). (2019) 98:e16211. 10.1097/MD.0000000000016211 31261573 PMC6617425

[10] Fonseca S Costa F Seabra M Dias R Soares A Dias C. Systemic inflammation status at admission affects the outcome of intracerebral hemorrhage by increasing perihematomal edema but not the hematoma growth. Acta Neurol Belg. (2021) 121:649–59. 10.1007/s13760-019-01269-2 31912444

[11] Ng LG Ostuni R Hidalgo A. Heterogeneity of neutrophils. Nat Rev Immunol. (2019) 19:255–65. 10.1038/s41577-019-0141-8 30816340

[12] Herrera VLM Walkey AJ Nguyen MQ Gromisch CM Mosaddhegi JZ Gromisch MS. A targetable ‘rogue' neutrophil-subset, [CD11b+DEspR+] immunotype, is associated with severity and mortality in acute respiratory distress syndrome (ARDS) and COVID-19-ARDS. Sci Rep. (2022) 12:5583. 10.1038/s41598-022-09343-1 35379853 PMC8977568

[13] Decano JL Viereck JC McKee AC Hamilton JA Ruiz-Opazo N Herrera VL. Early-life sodium exposure unmasks susceptibility to stroke in hyperlipidemic, hypertensive heterozygous Tg25 rats transgenic for human cholesteryl ester transfer protein. Circulation. (2009) 119:1501–9. 10.1161/CIRCULATIONAHA.108.833327 19273719 PMC2825876

[14] Herrera V Gromisch CM Decano JL Tan GAL Pasion KA Hua N Greer DM Ruiz-Opazo N. Anti-DEspR antibody treatment after spontaneous intracerebral hemorrhage (ICH) in ICH-prone Dahl salt-sensitive hypertensive rat model increases overall survival, while pre-emptive treatment delays ICH onset. Abstract 1467. Eur Soc Stroke. (2021) 6:90. 10.1177/23969873211034932 35252582 PMC8414986

[15] Wang XM Zhang YG Li AL Long ZH Wang D Li XX. Expressions of serum inflammatory cytokines and their relationship with cerebral edema in patients with acute basal ganglia hemorrhage. Eur Rev Med Pharmacol Sci. (2016) 20:2868–71. 27424987

[16] Ducruet AF Zacharia BE Hickman ZL Grobelny BT Yeh ML Sosunov SA. The complement cascade as a therapeutic target in intracerebral hemorrhage. Exp Neurol. (2009) 219:398–403. 10.1016/j.expneurol.2009.07.018 19632224 PMC3731062

[17] Zheng GR Chen B Shen J Qiu SZ Yin HM Mao W. Serum myeloperoxidase concentrations for outcome prediction in acute intracerebral hemorrhage. Clin Chim Acta. (2018) 487:330–6. 10.1016/j.cca.2018.10.026 30347182

[18] Alioglu Z Bülbül I Orem A Ozmenoglu M Vanizor B Boz C. Increased plasma endothelin-1 levels in patients with intracerebral hemorrhage. J Stroke Cerebrovasc Dis. (2000) 9:176–80. 10.1053/jscd.2000.7231 24192024

[19] Zouki C Baron C Fournier A Filep JG. Endothelin-1 enhances neutrophil adhesion to human coronary artery endothelial cells: role of ET(A) receptors and platelet-activating factor. Br J Pharmacol. (1999) 127:969–79. 10.1038/sj.bjp.0702593 10433505 PMC1566081

[20] Herrera VL Ponce LR Bagamasbad PD VanPelt BD Didishvili T Ruiz-Opazo N. Embryonic lethality in Dear gene-deficient mice: new player in angiogenesis. Physiol Genomics. (2005) 23:257–68. 10.1152/physiolgenomics.00144.2005 16293765

[21] Yousefi S Mihalache C Kozlowski E Schmid I Simon HU. Viable neutrophils release mitochondrial DNA to form neutrophil extracellular traps. Cell Death Differ. (2009) 16:1438–44. 10.1038/cdd.2009.96 19609275

[22] Urday S Kimberly WT Beslow LA Vortmeyer AO Selim MH Rosand J. Targeting secondary injury in intracerebral haemorrhage—perihaematomal oedema. Nat Rev Neurol. (2015) 11:111–22. 10.1038/nrneurol.2014.264 25623787

[23] Hemorrhagic Stroke Academia Industry (HEADS) Roundtable Participants & Second HEADS Roundtabled Participants. Recommendations for clinical trials in ICH: the second hemorrhagic stroke academia industry roundtable. Stroke. (2020) 51:1333–8. 10.1161/STROKEAHA.119.027882 32078490 PMC7093252

[24] de Bont CM Boelens WC Pruijn GJM. NETosis, complement, and coagulation: a triangular relationship. Cell Mol Immunol. (2019) 16:19–27. 10.1038/s41423-018-0024-0 29572545 PMC6318284

[25] Holste K Xia F Garton HJL Wan S Hua Y Keep RF. The role of complement in brain injury following intracerebral hemorrhage: a review. Exp Neurol. (2021) 340:113654. 10.1016/j.expneurol.2021.113654 33617886 PMC8119338

[26] Meegan JE Yang X Coleman DC Jannaway M Yuan SY. Neutrophil-mediated vascular barrier injury: role of neutrophil extracellular traps. Microcirculation. (2017) 24:12352. 10.1111/micc.12352 28120468 PMC5404986

[27] Giede-Jeppe A Bobinger T Gerner ST Sembill JA Sprügel MI Beuscher VD. Neutrophil-to-lymphocyte ratio is an independent predictor for in-hospital mortality in spontaneous intracerebral hemorrhage. Cerebrovasc Dis. (2017) 44:26–34. 10.1159/000468996 28419988

[28] Menon G Johnson SE Hegde A Rathod S Nayak R Nair R. Neutrophil to lymphocyte ratio – a novel prognostic marker following spontaneous intracerebral haemorrhage. Clin Neurol Neurosurg. (2021) 200:106339. 10.1016/j.clineuro.2020.106339 33183885

[29] Shi M Li XF Zhang TB Tang QW Peng M Zhao WY. Prognostic role of the neutrophil-to-lymphocyte ratio in intracerebral hemorrhage: a systematic review and meta-analysis. Front Neurosci. (2022) 16:825859. 10.3389/fnins.2022.825859 35360156 PMC8960242

[30] Chen W Wang X Liu F Tian Y Chen J Li G. The predictive role of postoperative neutrophil to lymphocyte ratio for 30-day mortality after intracerebral hematoma evacuation. World Neurosurg. (2020) 134:e631–5. 10.1016/j.wneu.2019.10.154 31682990

[31] Lattanzi S Cagnetti C Rinaldi C Angelocola S Provinciali L Silvestrini M. Neutrophil-to-lymphocyte ratio improves outcome prediction of acute intracerebral hemorrhage. J Neurol Sci. (2018) 387:98–102. 10.1016/j.jns.2018.01.038 29571881

[32] Lattanzi S Brigo F Trinka E Cagnetti C Di Napoli M Silvestrini M. Neutrophil-to-lymphocyte ratio in acute cerebral hemorrhage: a system review. Transl Stroke Res. (2019) 10:137–45. 10.1007/s12975-018-0649-4 30090954

[33] Greenberg SM Ziai WC Cordonnier C Dowlatshahi D Francis B Goldstein JN. American Heart Association/American Stroke Association. 2022 guideline for the management of patients with spontaneous intracerebral hemorrhage: a guideline from the American Heart Association/American Stroke Association. Stroke. (2022) 17:101161. 10.1161/STR.0000000000000407 35579034

[34] Eruslanov EB Singhal S Albelda SM. Mouse versus human neutrophils in cancer: a major knowledge gap. Trends Cancer. (2017) 3:149–60. 10.1016/j.trecan.2016.12.006 28718445 PMC5518602

[35] Hemorrhagic Stroke Academia Industry (HEADS) Roundtable Participants. Basic and translational research in intracerebral hemorrhage: limitations, priorities, and recommendations. Stroke. (2018) 49:1308–14. 10.1161/STROKEAHA.117.019539 29618555 PMC5915965

