# Supplementary material for: “Rogue” [DEspR+CD11b+] neutrophil subset correlates with severity in spontaneous intracerebral hemorrhage
Source: Front Neurol. 2022 Jul 25;13:935579. doi: 10.3389/fneur.2022.935579 (PMC9358208; doi:10.3389/fneur.2022.935579)
Supplement: Supplementary file 3 [file Presentation_1.pdf]

Supplementary Fig. S1

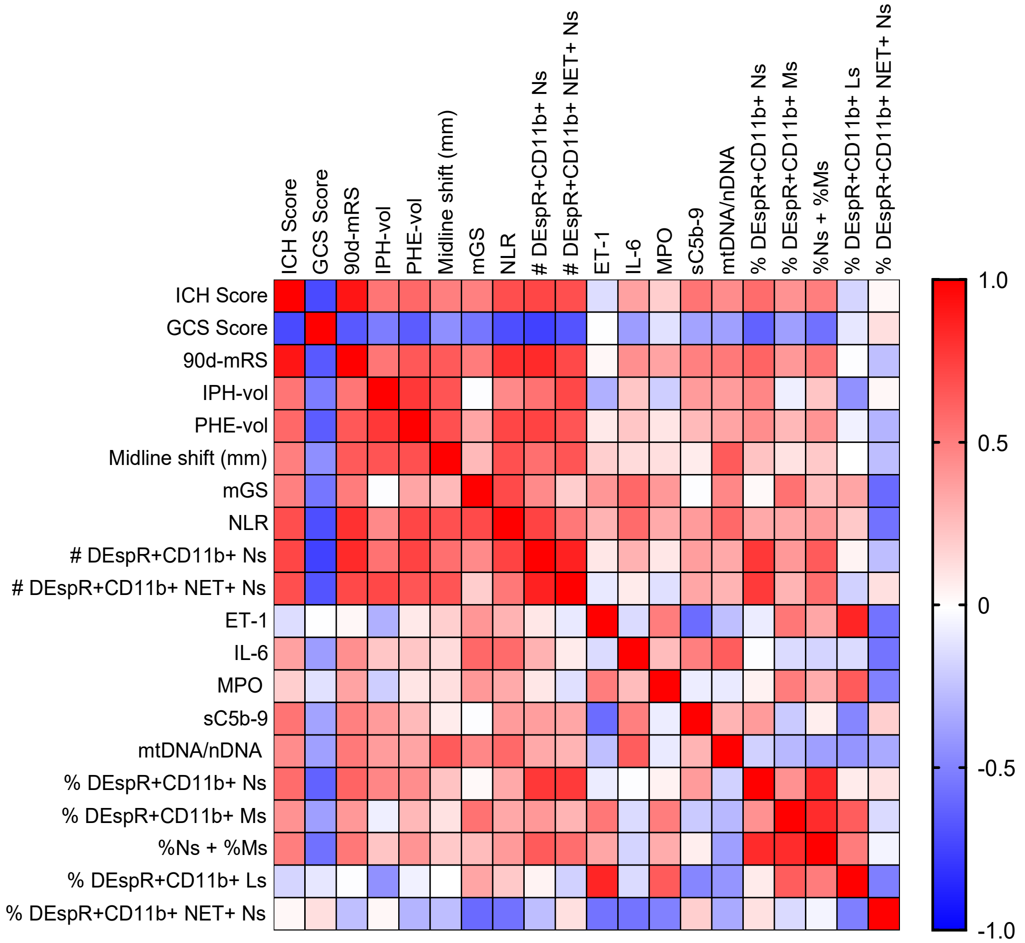

Supplementary Fig. S1. Correlation matrix analysis of clinical parameters, flow cytometry measures, and plasma biomarkers in sICH. Correlation matrix showing Spearman Rank Correlation range -1.0 to 1.0, n = 13 sICH patients admitted to the ICU. Parameters in correlation matrix analysis by category: 1] Clinical parameters: ICH-score on admission, GCS on day of FCM-analysis; 90-day modified Rankin Scale (mRS), neutrophil-lymphocyte ratio (NLR); 2] Radiological parameters on day of FCM-analysis: perihematomal edema (PHE)-vol and intraparenchymal hemorrhage (IPH)-vol (ml), midline shift (mm), modified Graeb score (mGS); 3] Flow cytometry parameters: cell surface DEspR+ expression levels (% or number # K/μL) on CD11b+ activated neutrophils (Ns), monocytes (Ms), and lymphocytes (Ls). 4] Plasma biomarkers reported to be elevated in sICH by others [interleukin-6 (IL-6), endothelin-1 (ET1), myeloperoxidase (MPO), terminal complex of complement (sC5b9), and number of copies of mitochondrial/nuclear DNA ratio (mt/nDNA). 5] NET-formation parameters: NET+Ns, NET-forming neutrophils; number (#) or % of circulating DEspR+CD11b+ NET-forming neutrophils. Data, Spearman correlation coefficients, 95% confidence intervals (CI) are listed in Supplementary Table S2.

Supplementary Table S3. Spearman rank correlation analysis of top-3 peripheral neutrophil-markers with clinical and radiological measures of sICH severity (n = 13).

	Clinical Measures			***Radiological PARAMETERS***	
	t-1 GCS Score	***Admission ICH Score***	***90d mRS***	***t-1 IPH-vol***	***t-1 PHE-vol***
MARKER	***r*** **[*p^B value*]**	***r*** **[*p^B value*]**	***r*** **[*p^B value*]**	***r*** **[*p^B value*]**	***r*** **[*p^B value*]**
**t-1 # DEspR+CD11b+ Ns (K/µL)**	**-0.75 [0.012^B]**	**0.72 [0.018^B]**	**0.83 [0.003^B]**	0.55 [n.s.]	**0.74 [0.018^B]**
**t-1 # DEspR+CD11b+NET+ Ns (K/µL)**	-0.67 [0.042^B]	0.69 [0.033^B]	**0.71 [0.024^B]**	**0.71 [0.024 ^{B}]**	0.67 [0.048^B]
**t-1 Neutrophil-to-Lymphocyte Ratio (NLR)**	-0.70 [0.030^B]	0.69 [0.030^B]	**0.81 [0.003^B]**	0.46 [n.s.]	**0.73 [0.021^B]**

LEGEND: Pilot Observational Study (single site) n = 13 subjects. Spearman Rank Order Correlation coefficient (*r*), with Bonferroni corrected *p* value x 3, p^B.

t-1, day of flow cytometry analysis; # DEspR+CD11b+ Ns: total number (#) in K/µL of DespR+C11b+ neutrophils (Ns); # DespR+CD11b+NETosing Ns: number of DespR+CD11b+ NET-forming Ns (% DespR+CD11b+ NET-forming Ns x total number of DespR+CD11b+ Ns); NLR: absolute neutrophil count/absolute lymphocyte ratio; GCS Score, Glasgow Coma Scale; ICH Score, admission intracerebral hemorrhage score; 90d-mRS, 90-day modified Ranking Scale-score; IPH-vol, intraparenchymal volume (ml); PHE-vol, perihematomal edema volume (ml).

r, Spearman Correlation coefficient *rho*; *rho* ≥ 0.71: $\alpha < 0.05$, power > 0.8 with n = 13 (bold red and boxed). Significant ($p < 0.05$) correlation, significant *r*, but power < 0.8 (blue font, not bold).
